# The Deposition of Hydroxyapatite Particles Within an Organic Matrix on the Surface of Poly(lactic acid)

**DOI:** 10.3390/ijms252111587

**Published:** 2024-10-29

**Authors:** Katarzyna Dopierała, Emilia Krok, Ewa Stachowska, Jagoda Nowak-Grzebyta, Krzysztof Walczak, Jacek Andrzejewski, Krystyna Prochaska

**Affiliations:** 1 Institute of Chemical Technology and Engineering, Poznan University of Technology, Berdychowo 4, 60-965 Poznan, Poland; katarzyna.dopierala@put.poznan.pl (K.D.); krzysztof.walczak@student.put.poznan.pl (K.W.); 2 Institute of Physics, Poznan University of Technology, Piotrowo 3, 60-965 Poznan, Poland; emilia.krok@put.poznan.pl; 3 Institute of Mechanical Technology, Poznan University of Technology, Piotrowo 3, 60-965 Poznan, Poland; ewa.stachowska@put.poznan.pl (E.S.); jagoda.pa.nowak@doctorate.put.poznan.pl (J.N.-G.); 4 Institute of Materials Technology, Poznan University of Technology, Piotrowo 3, 61-138 Poznan, Poland; jacek.andrzejewski@put.poznan.pl

**Keywords:** hydroxyapatite, poly(lactic acid), Langmuir–Blodgett, implant, surface modification

## Abstract

Hydroxyapatite (HAP) is a well-established material in biomedical applications, especially for bone tissue regeneration, dental implants, and drug delivery systems. Recent research emphasizes enhancing the biocompatibility and osteoconductivity of orthopedic implants using HAP. This study explores the potential of combining HAP with a lipid matrix to improve the surface properties and biocompatibility of poly(lactic acid) (PLA)-based, 3D-printed, resorbable bone implants. We utilized the Langmuir–Blodgett method to deposit HAP within a dihexadecyl phosphate (DHP) matrix onto PLA substrates. This study demonstrates that DHP and HAP form stable monolayers at the air/water interface with HAP particles distributed within a homogeneous lipid matrix. The presence of HAP and the resulting changes in surface free energy (SFE) are hypothesized to enhance the biocompatibility of PLA implants. Our findings indicate that films composed of DHP + HAP 5:1 are particularly effective in altering PLA surface characteristics, potentially improving osteointegration, and reducing microbial adherence. Overall, this work highlights that surface modification of PLA with HAP and lipid matrices is the first step towards new, promising, and cost-effective strategies for developing advanced biomaterials for bone regeneration.

## 1. Introduction

Hydroxyapatite (Ca_10_(PO_4_)_6_(OH)_2_, HAP) is a widely used and accepted material in the biomedical field. Current research on HAP spans various areas such as bone tissue regeneration, dental implants, and drug delivery systems [1,2,3]. Considerable attention is given to HAP-based materials with ongoing studies aimed at developing and improving orthopedic implants with enhanced biocompatibility and osteoconductivity [4,5,6]. Additionally, HAP is known for its non-toxic, non-inflammatory, and non-immunogenic properties and is therefore often incorporated into polymeric composites for bone tissue engineering applications [7,8]. Various techniques have been offered to modify the surface of orthopedic implants using HAP. Several coating techniques have been demonstrated to deposit HAP on titanium materials using either physical or chemical deposition [4]. Zinc, strontium, and other metal ions are often used to dope HAP particles and reduce the biofilm formation which is a serious problem in implant surgery leading to infections and additional, costly medical procedures [9].

Among several possible side effects of the surgery, foreign body reaction (FBR) is a response of the tissues and cells triggered by their exposure to foreign substances or materials, including implants, medical devices, stents, or various drug-releasing biomaterials [10]. Extensive research has been conducted to understand the importance of proteins in the fibrotic response of the body. These molecules undergo significant conformational changes upon adsorption to the implant surface, creating an environment conducive to macrophage and fibroblast attachment. However, recent studies emphasize that not only proteins may play a pivotal role in the immune response of the cells, as the process of fibrosis is also strongly dependent on the type of lipid molecules [11].

Lipids such as cholesterol, eicosanoids, sphingolipids, or lysophospholipids are considered critical molecules in various anti-inflammatory and pro-inflammatory pathways [12]. Glass surfaces coated with 16:0–18:1 phosphatidylethanolamine (POPE) exhibit osteoinductive properties which lead to substantial improvement in osseointegration, at the same time demonstrating antimicrobial properties against Gram-positive and Gram-negative bacterial strains [13]. Phosphatidylserine (PS) and phosphatidylinositol (PI), due to the negatively charged headgroups, easily associate with the positively charged calcium molecules [14]. These calcium-binding lipids deposited on the titanium alloy form a mineral matrix, thus creating a calcium- and phosphate-rich environment that promotes the process of osseointegration [15]. The calcium ions transferred from the aqueous calcium chloride solution to the titanium and stainless steel supports have also been successfully utilized by deposition of Langmuir monolayers composed of dihexadecyl phosphate (DHP) [16]. The homogeneous DHP lipid matrix was further used to induce the growth of carbonated hydroxyapatite, which is known to promote bone regeneration at the early post-implantation stages. The results presented in [16] indicated increased osteoblastic cell proliferation and improved cell viability. Undoubtedly, the use of lipid films, either in the form of pre-coatings or as cell growth-promoting matrices deposited on the implant surface, can significantly improve the overall biocompatibility of the biomaterial, increase its osseointegration potential, and consequently prevent the implant rejection by the patient’s organism. 

The above-mentioned reports demonstrate that the biocompatibility of polymeric implants can be partially controlled by a proper surface treatment of the material. The presence of functional groups or charge, surface degradation, hydrophilic–hydrophobic properties, wettability, and surface free energy, as well as roughness, stiffness, and protein binding are targets of interest for researchers aiming at the development of biomedical materials [17].

In our previous work, we demonstrated the strategy for surface modification of poly(lactic acid) using hydroxyapatite particles using the Langmuir–Schaefer method [18]. The presence of HAP altered the topography and roughness of PLA at the nanoscale, which can be beneficial for the osteointegration of polymeric implants and a reduction in microbial adherence. 

Due to its physicochemical properties, PLA is one of the most popular materials used in the 3D printing industry [19]. In this case, the main characteristics that have made it popular are its low melting point (≈160 °C) and its minor shrinkage. The low shrinkage allows for the production of precise products without the risk of warping, which is important during the production of accurate parts. In addition to the benefits of favorable processing properties, the use of PLA in the biomedical industry is primarily related to the degradation ability of this polymer. The properties of PLA make it an excellent material for the production of personalized geometric structures, the application of which may include the production of bone implants. In the case of such a perspective, 3D printing allows the production of partially porous structures, where the ability to penetrate through liquid media facilitates the colonization of the implant structure by the human body’s osteogenic cells. The combination of the favorable macrostructural properties of PLA-based implants and the functionalization of their surface may constitute a further step in the development of synthetic bone-forming materials [20].

The objective of this work is to deposit HAP particles within a lipid matrix built of DHP on the surface of poly(lactic acid) to improve the surface properties and biocompatibility of the polymeric, 3D-printed, resorbable bone implant. It is expected that the combination of HAP and DHP will affect the surface free energy of the material and improve its biocompatibility.

## 2. Results and Discussion

### 2.1. Proliferation and Cytotoxicity Studies of 3D-Printed PLA

**Figure 1:**
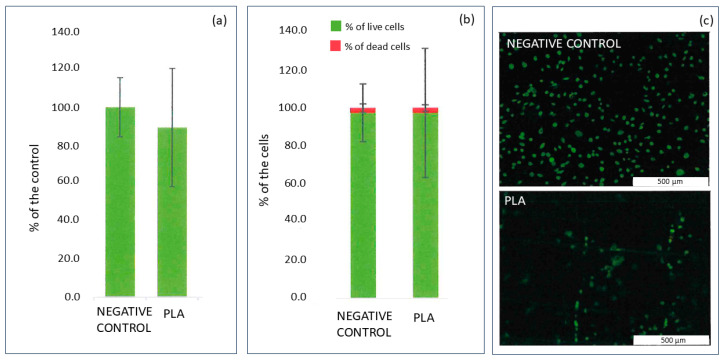
The evaluation of cell proliferation after 48 h of direct contact with PLA material (**a**), the results of the live/dead test showing the cell viability after 48 h of direct contact with PLA sample (**b**), and the selected microscope images taken during the live/dead test (**c**). The green color on the images represents live cells; the red color (almost invisible here) represents the dead cells.

The proliferation and cytotoxicity studies were performed on PLA samples before the deposition of the coating and the results are presented in Figure 1. The microscope images were analyzed in terms of Saos-2 cell morphology, as well as the number of live and dead cells. The results in Figure 1a are presented as % of negative control, while Figure 1b shows the ratio of live cells to dead cells. Figure 1c shows the selected images taken for negative control and for PLA samples. For the PLA sample, significant adhesion of the cells to the material was observed, which is a positive effect in terms of application as a resorbable implant. The uneven distribution of the cells on the surface of the PLA sample was noticed as a result of the 3D-printed sample’s structure. This results in large values of standard deviations in Figure 1a,b. Overall the PLA sample promotes cell growth, which is a promising result for potential tissue regeneration.

### 2.2. HAP-DHP Monolayers at the Air/Water Interface

**Figure 2:**
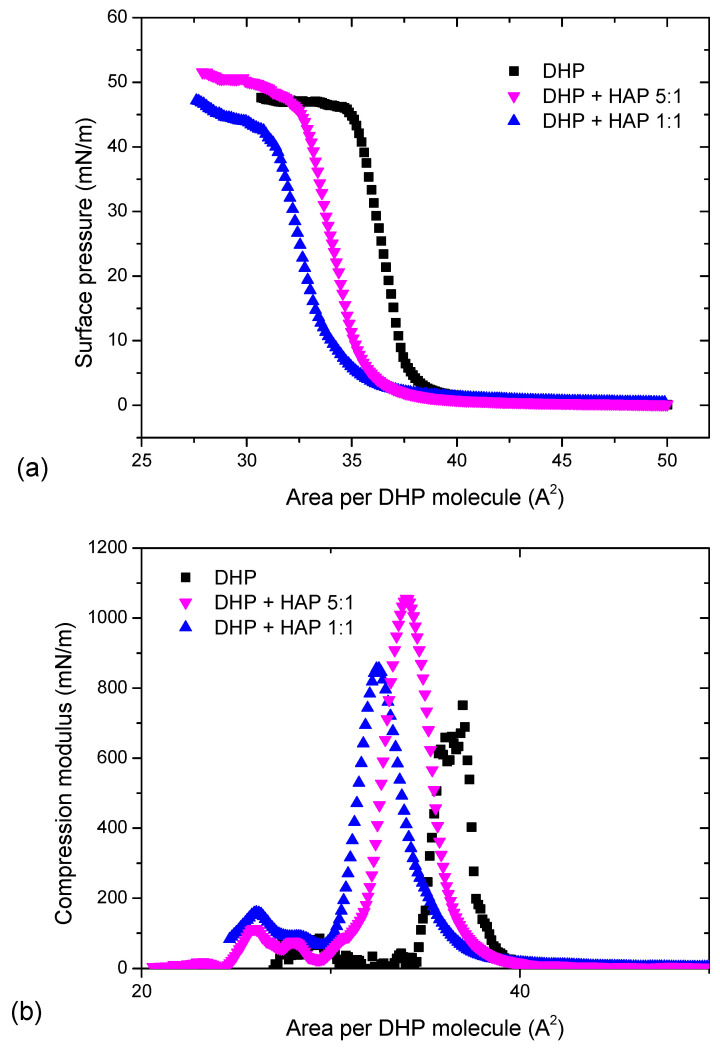
The π-A isotherms (**a**) and compression modulus curves (**b**) for DHP and DHP + HAP monolayers formed at the air/water interface at various ratios of DHP to HAP.

**Figure 3:**
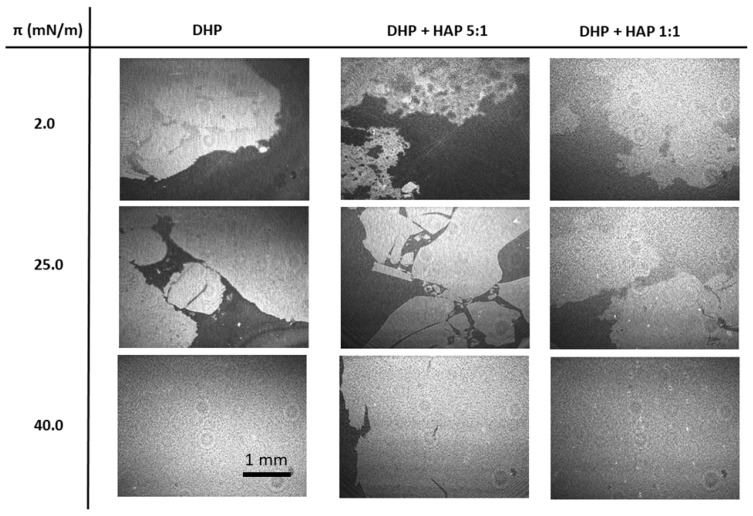
BAM images of DHP monolayers in the presence of various amounts of HAP particles.

The compression of dihexadecyl phosphate molecules leads to an increase in the surface pressure to 48 mN/m, followed by a collapse of the monolayer at molecular area 36 Å^2^/molec., as shown in Figure 2a. The presence of DHP particles at the interface leads to the shift of the curve towards smaller areas occupied by a single DHP molecule. In the case of DHP + HAP 5:1, the monolayer collapses at a slightly higher surface pressure, while for the DHP + HAP 1:1, the monolayer loses its two-dimensional structure at a lower surface pressure. Hydroxyapatite particles make the film less fluid as the compression modulus, presented in Figure 2b, reaches larger values for mixed films than for pure DHP. The course of the isotherms suggests the presence of both components at the interface. The HAP particles dispersed in the organic film are visualized in BAM images in Figure 3. The expanded organic films at a low surface pressure can be observed in all images captured at π = 2.0 mN/m. Further compression leads to the formation of condensed films. The HAP particles are visible in the BAM images for the condensed DHP + HAP 1:1 film. The morphology of the film suggests a random distribution of hydroxyapatite particles in the organic matrix without a clear manifestation of aggregation. The additional stability studies (see Figure S1 in Supporting Information) proved the improved stability of two-component monolayers at the 1:1 and 5:1 ratios in comparison to pure HAP film.

### 2.3. Characterization of the Modified PLA

**Figure 4:**
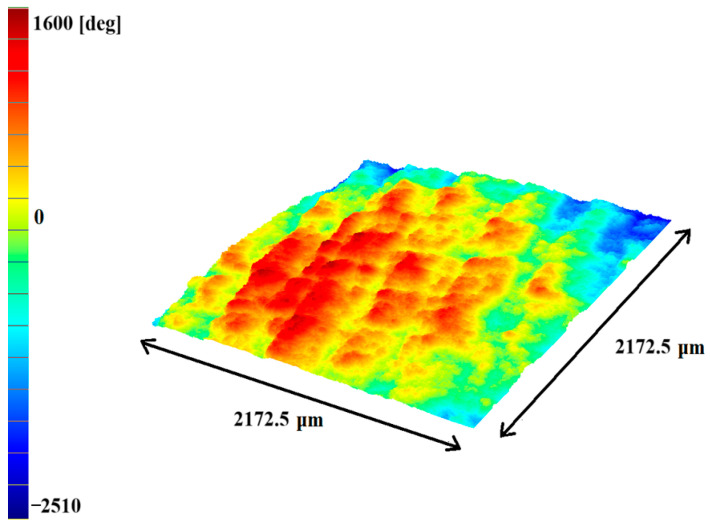
A computer 3D reconstruction of the holographic unwrapped phase image of the area of the PLA plate surface (microscope magnification 2.5×). The values in degrees on the Y axis correspond to the phase change of the laser beam after passing through the sample.

The ability to determine surface metrological parameters using a DHM microscope was used to verify the quality and repeatability of sample preparation via 3D printing; this is presented in Figure 4 for printed PLA samples. Four samples each with dimensions of 15 × 15 × 0.2 mm were tested. For each plate, 30 areas on the surface with dimensions of 2.17 × 2.17 mm^2^ were selected and the average waviness parameter *W_a_* and the average roughness parameter *S_a_* were determined. In general, waviness parameters refer to a larger component of surface texture on which roughness is overlayed. In our case, the repeatability of *W_a_* values reflected the repeatability of the filament arrangement. The average *W_a_* values ranged from 5.3 µm to 6.3 µm and the standard deviations did not exceed 10%. The average roughness parameter *S_a_* refers to the surface roughness and heterogeneity of the PLA density in the filament. The mean values of the *S_a_* parameter differed only slightly from *W_a_* (4.7–6.3 µm) and were characterized by similar repeatability with a standard deviation of 7%. Moreover, it was noticed that the values of the above parameters for the sample surface adjacent to the heating plate of the 3D printer were approximately 20% smaller. The obtained results confirmed the appropriate surface quality of the printed PLA samples. Figure 4 shows an example of a computer 3D reconstruction of the holographic unwrapped phase image for one of the measured areas.

**Figure 5:**
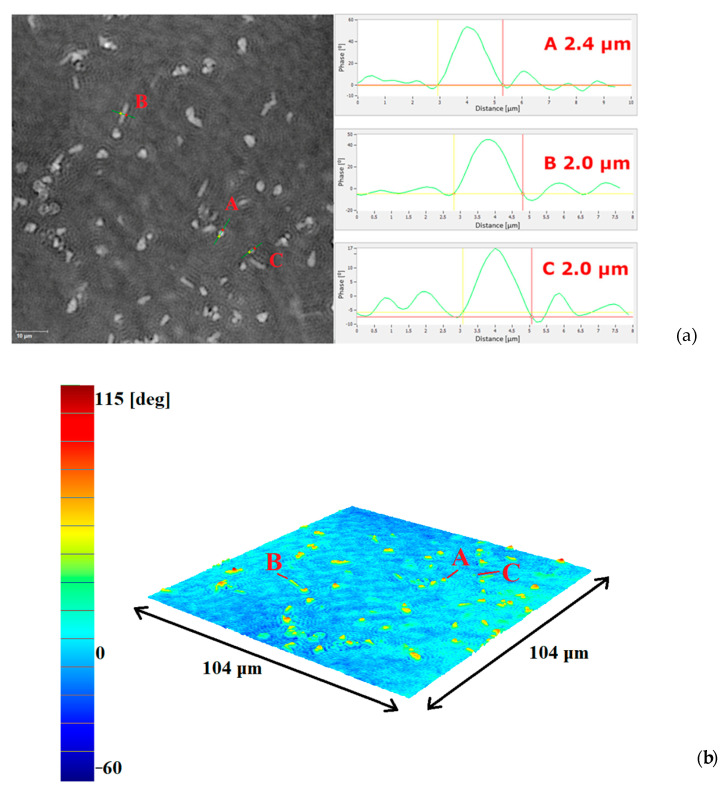
Holographic unwrapped phase image and corresponding optical cross-section profiles of HAP particles (microscope magnification 50×) with area 104 × 104 µm^2^ (**a**) and computer 3D reconstruction of holographic unwrapped phase image (microscope magnification 50×). Values in degrees on Y axis correspond to phase change of laser beam after passing through sample (**b**). The letters A, B and C in Figure 5b correspond to profiles shown in Figure 5a.

The effect of DHP + HAP 1:1 deposition was also examined using a DHM T1000 microscope with 50× magnification. The observation of HAP particles in the DHP matrix gave a similar image to the HAP powder scattered on a microscope slide (see Figure S2 in Supplementary Material), whereas the molecules of DHP formed a rather optically homogeneous background. Therefore, in the phase image and in the computer 3D reconstruction image, only HAP particles are visible (see Figure 5). The profiles A, B, and C are similar to those obtained for the HAP on the glass substrate. The diameters of visible HAP particles are approximately 2–2.5 µm. Some of the particles formed small agglomerates, the largest size of which did not exceed 8 µm. The phase change (about 60 deg) of the laser beam passing through the HAP particles and the DHP-matrix is slightly lower than for HAP on glass (see Supplementary Material Figure S2), which confirms the presence of DHP in the sample with a slightly lower refractive index than for glass (n_DHP_ = 1.489).

On this basis, we concluded that the DHP + HAP 1:1 layer was effectively deposited on the substrate using the LB method.

**Figure 6:**
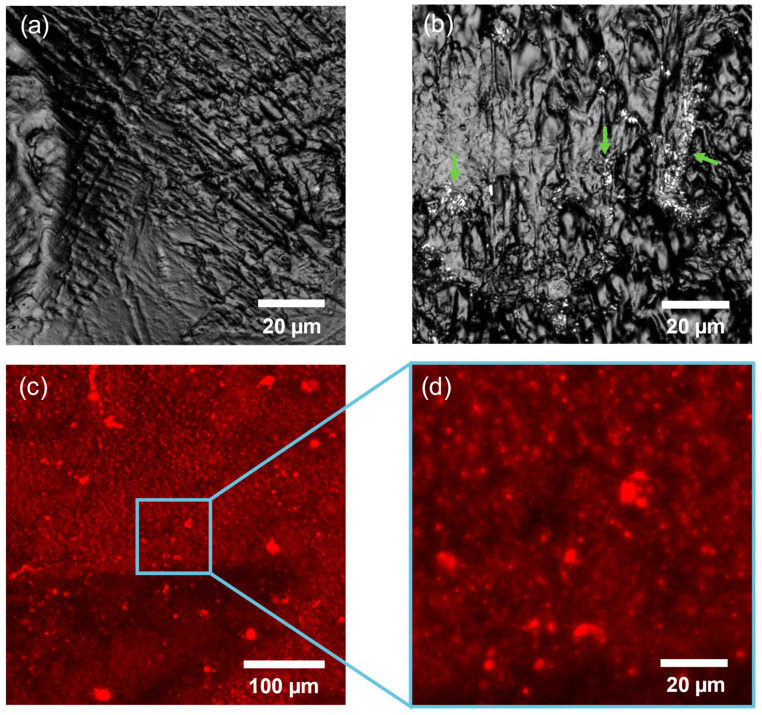
Confocal images of DHP-HAP 1:1 monolayer deposited on PLA substrate: (**a**) PLA surface without deposited DHP-HAP monolayer; (**b**) PLA surface with marked HAP aggregates—green arrows point to local agglomerates of HAP; (**c**) fluorescence image of DHP monolayer, labeled with DOPE-Atto 633—the blue square indicates the magnified area in panel (**d**).

Confocal imaging was carried out to verify the successful transfer of the DHP-HAP 1:1 monolayer on the surface of the PLA support. Comparison of the untreated surface (Figure 6a) with the sample exposed to one cycle of DHP-HAP deposition (Figure 6b) revealed the presence of the local agglomerates of HAP immobilized on top of the PLA substrate. As shown in our previous study, the diameter of the individual HAP nanoparticle was estimated to be around 200 ± 2.5 nm, according to dynamic light scattering measurements [18]. It is crucial to acknowledge that the resolution of the confocal microscope is constrained by the diffraction limit, which precludes resolving structures smaller than ~250 nm [21]. Consequently, only larger HAP aggregates were detectable in that instance, elucidating the relatively modest coverage of the PLA surface.

To demonstrate the deposition of the lipid matrix, the DHP solution was mixed with the widely employed fluorescent probe DOPE Atto-633, which has been effectively applied in visualizing supported lipid bilayers [22]. The addition of 0.1 mol% of the fluorescently labeled lipid, which was utilized in this research, does not alter the fundamental properties of the lipid film; instead, it facilitates lipids visualization under a fluorescence microscope. The captured images, measuring 212 × 212 µm in size, confirmed the formation of a uniformly deposited lipid monolayer (Figure 6c,d). Importantly, the lipid film also covered substrate grooves, which were material features resulting from the applied PLA printing technique. The visible, brighter features deposited on top of the PLA most likely corresponded to the HAP-DHP agglomerates, which were also visible in the bright field imaging. Overall, the performed confocal imaging confirmed the successful transfer of the lipid matrix, together with the embedded HAP nanoparticles.

The surface free energy of PLA before and after the deposition of LB film was determined using van Oss–Good and OWRK methods. The SFE values shown in Table 1 for untreated PLA were equal to 50.8 and 54.3 mJ/m^2^ based on the van Oss–Good and OWRK protocols, respectively. These values are in agreement with our previously reported data for 3D-printed material [18]; however, there is a noticeable difference between the wettability and surface free energy of PLA fabricated by various methods. For instance, the injection-molded PLA is characterized by SFE equal to 37.1 mJ/m^2^ (OWRK model), according to Vilaplana et al. [22]. These discrepancies result from roughness being affected during the 3D printing process. The SFE values for PLA after deposition of the DHP + HAP layer are generally smaller than before surface treatment, except for five layers of DHP + HAP 5:1 deposited on PLA, which induced an increase in SFE to 53.4, according to the OWRK model. This value is similar to the result reported earlier for plasma-treated PLA [23]. The data obtained from the OWRK model indicates a significant contribution of the dispersive part ($\gamma_{s}^{d}$), almost irrespective of the number of the transferred LB layers and the DHP to HAP ratio. On the other hand, the amount of HAP particles in the film affected the polar part ($\gamma_{s}^{p}$), varying between 3.3 mJ/m^2^ for a single layer of DHP + HAP 1:1 and 20.0 mJ/m^2^ for five layers of DHP + HAP 5:1.

The long-range interactions represented by the large $\gamma_{s}^{LW}$ values contribute significantly to SFE value in the van Oss–Good model. Comparing the values of acid–base components, we observe a notable difference between DHP + HAP 5:1 (5 L) and the rest of the samples. The short-range interactions affect that sample more than any other surface listed in Table 1, which might be explained by the forces controlling the architecture of multilayer formed by DHP + HAP 5:1. Similarly to the OWRK model, the SFE values increase with the number of monolayers deposited on PLA. This can be explained by the increasing surface coverage with each subsequent stroke. However, the results shown in Table 1 suggest that at least five deposited layers of DHP + HAP with domination of the lipid component are necessary to increase the surface free energy of PLA. Yet, considering the low amount of material consumed to fabricate the coating, the LB technique is an effective and low-cost approach.

In contrast to our previous work, in which pure HAP was used as the coating material, here we used HAP together with lipids, which enabled vertical deposition [18]. This means we could deposit the coating on both sides of the implant in a single step. Moreover, we have shown lipid coating is a versatile strategy to load various numbers of nanoparticles, depending on the goal. In comparison to other strategies of surface modification, such as electrospinning or layer-by-layer, the method we presented in this work is cost-effective and requires limited amounts of lipids and HAP particles [18,24].

## 3. Materials and Methods

### 3.1. Materials 

Polylactide-based unmodified filament, with a diameter of 1.75 mm, supplied by the company PlastSpaw (Lubliniec, Poland), was utilized. Commercially available synthetic hydroxyapatite with a chemical formula of [Ca_5_(OH)(PO_4_)_3_]_x_ was kindly supplied by Syntplant sp. z o.o. (Poznań, Poland) in a form of whitish powder with a defined elemental composition (CaO 54.22, P_2_O_5_ 33.05, and MgO 0.11%m/m). Dihexadecyl phosphate (DHP) and 1,2-dioleoyl-sn-glycero-3-phosphoethanolamine labeled with the fluorescent dye Atto 633 (DOPE-Atto 633) were purchased from Merck KGaA, Darmstadt, Germany, and dissolved in chloroform of spectroscopic grade (Uvasol, Merck). Absolute ethanol was used as the solvent for the preparation of HAP dispersion. For proliferation and cytotoxicity studies, we used phosphate buffer saline, McCoy’s 5A cell culture medium, and fetal bovine serum purchased from Capricorn. Penicillin–Streptomycin–Neomycin (P/S/N) was purchased from Merck. The 0.25% trypsin was supplied by Biowest (Nuaillé, France). Ethidium bromide homodimer and calcein-AM were purchased from Biotum (Fremont, CA, USA). The cell line Saos-2, similar to human osteoblasts, was purchased from ATTC (Brendale, Australia). The cell line was isolated and characterized by Fogh and Trempe [24].

### 3.2. Preparation of PLA Substrates

The 3D-printed polylactide specimens were prepared using the material extrusion method (MEX), also called FDM (fused deposition modeling). The manufacturing process was performed using the MK3S model from the company PrusaResearch (Prague, Czech Republic). The printer was equipped with a 0.4 mm brass nozzle. The table surface, which was covered with a layer of PEI (polyetherimide) film, was not further modified with adhesives. The thermal parameters of the printing process included the temperature of the nozzle and the working table, where the set point was 215 °C and 60 °C for the nozzle and table, respectively. In the present study, a commercially available filament was used, so the procedure did not require any additional process of extruding the material from the pellet. Prior to the process, the supplied 1.75 mm diameter filament was dried in a cabinet dryer (8 h, 60 °C).

**Figure 7:**
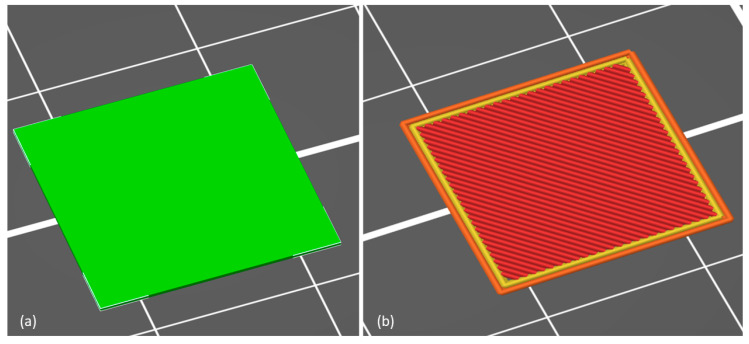
The procedure of the machine code preparation using the slicing software: (**a**) the view of the plate specimen 3D model; (**b**) the g-code simulation prepared before the printing process. The red lines represent the infill layers, while the yellow/orange lines associated with the shell layers of the specimen.

The sample preparation was conducted using the standard procedure, where the STL file was converted into machine code (g-code) using PrusaSlicer 2.5.0 software. The printing speed for the peripheral layers was 45 mm/s, while the printing of the fill area layers was carried out at 80 mm/s, with all speeds reduced to 20 mm/s for the first printing layer. The thickness of a single sample was only 0.2 mm, so the height of a single layer was reduced to 0.1 mm. The appearance of the model for a 15 × 15 × 0.2mm sample is shown in Figure 7; the picture also presents the simulation of the filament line placement (g-code). The 3D printing procedure was performed in batches of 30 pieces, where the printing process time was 24 min.

### 3.3. Proliferation and Cytotoxicity Studies

Saos-2 cells were stored in liquid nitrogen vapors. After thawing in a water bath, the cells were transferred to a 75 cm^3^ culture flask, and McCoy’s 5A medium supplemented with 15% FBS, 1.5 mM L-glutamine, and a 5% mixture of the antibiotic solution containing 5000 units/mL penicillin, 5 mg/mL streptomycin, and 10 mg/mL neomycin was added. The culture vessels were placed in an incubator under aseptic conditions. The culture was maintained under standard conditions, ensuring a constant temperature of 37 °C, 95% relative humidity, and 5% carbon dioxide content in the atmosphere. The culture medium was replaced every 48 h, and when the culture reached 80% confluency, the cells were passed to new culture vessels. Passaging was performed using 0.25% trypsin.

The proliferation of the Saos-2 cell line in contact with PLA samples was evaluated in live/dead assay. The cells (60,000 cells/mL) were seeded on the surface of sterile samples. The PLA samples were sterilized in UV-C for 15 min. The incubation lasted 48 h. Afterwards, the cells were stained with two dyes: calcein-AM for live cells, and ethidium homodimer-1 for dead cells. Live cells are identified by intracellular esterases, which catalyze the conversion of Calcein-AM to intensely fluorescent calcein, retained in the cytoplasm and emitting uniform green fluorescence. Ethidium homodimer-1, on the other hand, penetrates cells with damaged membrane integrity (dead cells) and forms a complex with DNA, exhibiting bright red fluorescence. Fluorescently labeled cells were observed using an Olympus GX 71 fluorescence microscope (Olympus, Tokyo, Japan) equipped with fluorescent filters, allowing the detection of signals from both live and dead cells. Saos-3 cells that had been seeded in a test plate and cultured in a full McCoy’s 5A medium without exposure to any harmful factors were used as a negative control. The measurements were performed over two independent repetitions.

### 3.4. A Monolayer Study

For monolayer formation, the commercial Langmuir trough (KSV Nima, Helsinki, Finland) with a surface area of 273 cm^2^ was used. The ultrapure water (18 MΩ·cm, pH 6.20, TOC 1–3 ppb) from a two-step purification process (DEMIWA 5 filtration system and ELGA PureLab Classic UV, Elga Veolia, Lane End, UK) was used as a subphase in all experiments. The temperature of 21 ± 1 °C was kept constant during the monolayer experiment using a thermostat connected to the trough. Just before the experiment, the hydroxyapatite dispersion (1 mg/mL) in absolute ethanol was filtered through a syringe filter of 0.22 µm pore size and sonicated. Afterwards, the dispersion was mixed with DHP solution at 1:1 and 5:1 volume ratios. The monolayer was formed by carefully spreading small droplets of DHP + HAP mixture on the subphase. After 10 min, the film was compressed using symmetrical barriers at a rate of 10 mm/min. The surface pressure (π) was measured by the Wilhelmy method using a platinum plate as a sensor. The accuracy and the resolution of measurements were 0.1 mN/m and 4 μN/m, respectively. The obtained isotherms were plotted as the surface pressure (π) versus area per lipid molecule (A). Each experiment was performed several times to ensure the reproducibility of 3 Å.

### 3.5. Brewster Angle Microscopy (BAM)

A Brewster angle microscope (microBAM, KSV Nima, Helsinki, Finland) was used to study the morphology of the films spread at the air/water interface. The equipment was coupled to the trough. A black plate was immersed in a subphase to absorb the refracted beam. The camera operated at a resolution of approximately 6 microns per pixel and captured images for the 3.6 to 4.0 mm field of view. The images were recorded simultaneously with the compression of the film at various surface pressures.

### 3.6. Deposition and Characterization of Langmuir–Blodgett Films

The films were deposited on 3D-printed PLA samples with dimensions of 15 × 15 mm. All samples were cleaned with isopropanol before applying the coating. The deposition rate was 2 mm/min and the transfer started with an upstroke. After the deposition, the substrate was dried and further characterized. In the case of multilayer transfer, the sample was left to dry for 20 min after each upstroke.

### 3.7. Confocal Microscopy

Confocal laser scanning microscopy (CLSM) images of the PLA surface with a deposited layer of HAP were obtained using LSM710 microscope (Zeiss, Jena, Germany) in reflected light mode, using EC Epiplan-Neofluar 20×, 0.5 air objective. For images obtained in white light, the excitation wavelength was 543 nm, while emission was collected in the wavelength range of 519–597 nm. Confocal images of the fluorescently labeled DHP + HAP monolayer deposited on PLA were obtained by using the excitation light from a He–Ne laser at 633 nm. Emission was collected in the wavelength range of 645–797 nm for the red light channel (Atto 633 detection range). All images were recorded as z-stack and merged using maximum intensity projection mode in Zeiss ZEN Blue software version 3.7 (Carl Zeiss Microscopy GmbH, Jena, Germany). Further adjustments of intensity and post-processing were carried out in Fiji/Image software version 1.54i (National Institutes of Health, Bethesda, Maryland, U.S.) [25,26].

### 3.8. Contact Angle Measurements and Determination of Surface Free Energy

The contact angle measurements were performed for PLA samples before and after the modification step. This was accomplished with a Theta Lite instrument (Biolin Scientific, Helsinki, Finland) working in a sessile drop mode. The contact angles were measured by releasing 3 μL of three probe liquids (water, diiodomethane, and formamide) onto the surface. Three drops were applied to each sample using an automated dispenser controlled by One Attension software (version 4.1.4, Biolin Scientific, Helsinki, Finland), and the average values were determined. The surface free energy (SFE) was calculated using the Owens, Wendt, Rabel, and Kaelble method (OWRK) and the van Oss–Good approach. The OWRK approach uses the contact angle data for two different liquids and it allows for distinguishing the polar and the dispersive parts of SFE according to Equation (1):

(1) $$\left(1+cos\theta_{i}\right)\gamma_{li}=2\left(\sqrt{\gamma_{li}^{d}\gamma_{s}^{d}}+\sqrt{\gamma_{li}^{p}\gamma_{s}^{p}}\right),$$

in which *γ_li_* is the surface tension of the probe liquid, *γ_s_* is the surface free energy of the solid substrate, and *d* is its dispersive and *p*—polar component [27]. The dispersion component is affected by the intermolecular interactions represented by London forces, while the hydrogen, acid–base, and inductive forces contribute to the polar part.

For comparison purposes, the SFE was determined using the van Oss–Good approach [28]. The contact angle data for three probe liquids: water, diiodomethane, and formamide were substituted into Equations (2) and (3):

(2) $$\left(1+cos\theta_{i}\right)\gamma_{li}=2$$

(3) $$\gamma =\gamma^{LW}+\gamma^{AB}=\gamma^{LW}+2\sqrt{\gamma^{+\gamma^{-}}}$$

In the van Oss–Good approach, the SFE is the sum of long-range interactions (LW–Lifshitz–van der Waals) and short-range, acid-base interactions (AB), mainly hydrogen bonds. The value of *γ^AB^* represents the geometric mean of the electron-donor and the electron-acceptor parameter. The value of *γ*^+^ is a base component that describes the ability of a surface to interact with the liquid capable of receiving electrons. Similarly, *γ*^+^ is an acid parameter characterizing the interactions of the surface and the liquid that can be an electron donor.

### 3.9. Digital Holographic Microscopy (DHM)

The microstructure and surface texture of the tested objects were examined using a DHM T1000 digital holographic transmission microscope (Lyncée tec, Lausanne, Switzerland) with off-axis geometry. Due to the very low illumination power of 200 µm/cm^2^ (light source—single-mode stabilized 666 nm laser with a linearly polarized beam), the tests were non-destructive. Two magnifications were used: 2.5× (objective NA = 0.07; FOV = max 2640 µm; no immersion) and 50× (objective NA = 0.75; FOV = max 132 µm; no immersion). The holograms were recorded using a CCD camera (1024 × 1024 pixels). The axial resolution was below 1 nm while the lateral resolution was 2.8 µm and 0.14 µm for 2.5× and 50× magnification, respectively. The three-dimensional reconstruction of the recorded phase images was performed using the Koala software standard (version 2014) (Lyncée Tec., Lousanne, Switzerland). Unwrapped phase images and their 3D reconstructions were then analyzed.

## 4. Conclusions

In this work, we demonstrated a possible path to modify PLA’s surface using two-component monolayers. Based on our results, the following conclusions might be formulated:

The coating materials, i.e., dihexadecyl phosphate and hydroxyapatite particles, can form a stable, two-component monolayer at the air/water interface. The particles at the interface are randomly distributed within an organic, homogenous matrix. No phase separation was induced when HAP was introduced into the film; however, the mixture with the excess of DHP was found to be more stable.

Our results show that DHP + HAP monolayers may be effectively deposited on the surface of poly(lactic acid) and change its surface characteristics, including surface free energy. The mixture with the domination of organic components was more effective in the alteration of surface free energy of PLA when compared to DHP:HAP 1:1. The presence of HAP and the increase in SFE induced by multilayers of DHP + HAP 5:1 might be beneficial for the biocompatibility of the polymeric implant.

A combination of lipids with hydroxyapatite allows for the preparation of stable film that can be deposited on both sides of an implant in a single step. The material we prepared by 3D printing promotes cell growth and is not cytotoxic for the Saos-3 cell line. Therefore, PLA combined with biomimetic coating based on hydroxyapatite and lipid may be considered as potentially interesting material for bone tissue regeneration.

As a general conclusion, it is important to remark that the deposition of LB film on 3D-printed PLA is a reasonable and economical method to modify the surface properties of polymeric material for potential biomedical applications.

## Figures and Tables

**Table 1:** The surface free energy values and its components determined by the van Oss–Good approach and OWRK method using the contact angle data for pure PLA, PLA coated with a single DHP monolayer, and two-component DHP + HAP films.

Sample	Van Oss–Good					OWRK		
	SFE (mJ/m^2^)	$\gamma_{s}^{LW}$	$\gamma_{s}^{AB}$	$\sqrt{\gamma_{s}^{+}}$	$\sqrt{\gamma_{s}^{-}}$	SFE (mJ/m^2^)	$\gamma_{s}^{d}$	$\gamma_{s}^{p}$
PLA	50.8	41.6	9.2	0.9	4.7	54.3	41.6	12.7
DHP	37.7	27.4	10.3	1.3	3.7	44.3	33.6	10.7
DHP + HAP 5:1 (1 L)	38.3	30.1	8.1	0.8	4.9	46.9	30.1	16.7
DHP + HAP 5:1 (3 L)	40.7	33.8	6.8	1.08	3.1	42.9	33.8	9.05
DHP + HAP 5:1 (5 L)	53.4	34.1	19.3	3.6	2.6	54.1	34.1	20.0
DHP + HAP 1:1 (1 L)	34.1	31.4	2.6	1.8	0.7	34.7	31.4	3.3

## Data Availability

The original contributions presented in the study are included in the article/Supplementary Material, further inquiries can be directed to the corresponding author.

## Supplementary Materials

The following supporting information can be downloaded at: https://www.mdpi.com/article/10.3390/ijms252111587/s1, Additional data from the monolayer study and images from the holographic digital microscope.
