# Supplementary material for: The Deposition of Hydroxyapatite Particles Within an Organic Matrix on the Surface of Poly(lactic acid)
Source: Int J Mol Sci. 2024 Oct 29;25(21):11587. doi: 10.3390/ijms252111587 (PMC11546348; doi:10.3390/ijms252111587)
Supplement: Supplementary file 1 [file ijms-25-11587-s001.zip › ijms-3221443-supplementary.pdf]

The deposition of hydroxyapatite particles within an organic matrix on the surface of poly(lactic) acid

Katarzyna Dopierała[1], Emilia Krok[3], Ewa Stachowska[3], Jagoda Nowak-Grzebyta[3], Krzysztof Walczak[1], Jacek Andrzejewski[4], Krystyna Prochaska*[1]

1- Institute of Chemical Technology and Engineering, Poznan University of Technology, Berdychowo 4, 60-965 Poznań, Poland

2- Institute of Physics, Poznan University of Technology, Piotrowo 3, 60-965 Poznan, Poland

3- Institute of Mechanical Technology, Poznan University of Technology, Piotrowo 3, 60-965 Poznan, Poland

4- Institute of Material Technology, Poznan University of Technology, Piotrowo 3, 61-138 Poznań, Poland

*corresponding author, e-mail: krystyna.prochaska@put.poznan.pl

Supporting information

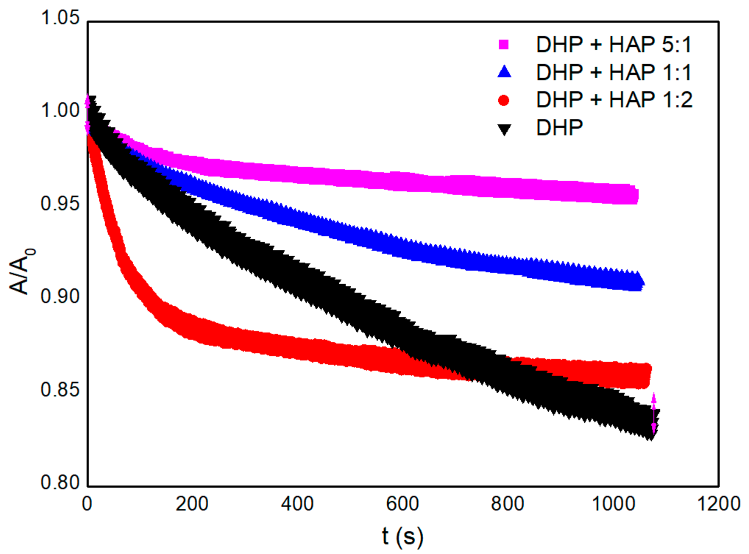

Figure S1 The relative area (A/A_0) changes over time measured for the DHP+HAP monolayers compressed to π=40 mN/m

The stability tests were performed for the DHP:HAP monolayers at various ratio of components. The films were compressed to π=40 mN/m and left to relax. The response of the

films was recorded as molecular area changes over time. For better visualization of the results, the data were shown as A/A_0 where A represents the mean molecular area at time t, while A0 represents the mean molecular area at time t=0s i.e. when the monolayer was compressed to target surface pressure. Figure S1 demonstrates the gradual loss of the monolayer material over time. The decrease of A/A_0 is the most intensive for DHP however it can be observed that for the monolayer at 5:1 ratio, the stability of the film is satisfactory since the loss of the material is less than 5%.

To identify HAP particles in DHM microscope images first a HAP-nano-powder was strewn on a microscope slide. In the phase image of the DHM microscope, you can see the powder grouped into spherical agglomerates (see Figure S2A). The profile along the green line (see Figure S2A,B) indicates that the smaller of them were about 2.5-3 µm in diameter and they caused a phase shift of about 70 deg when the laser light passed through. Computer reconstruction of the phase image (Figure S2C) shows also larger clusters of agglomerates for which the phase difference reached more than 400 deg.

A) B)

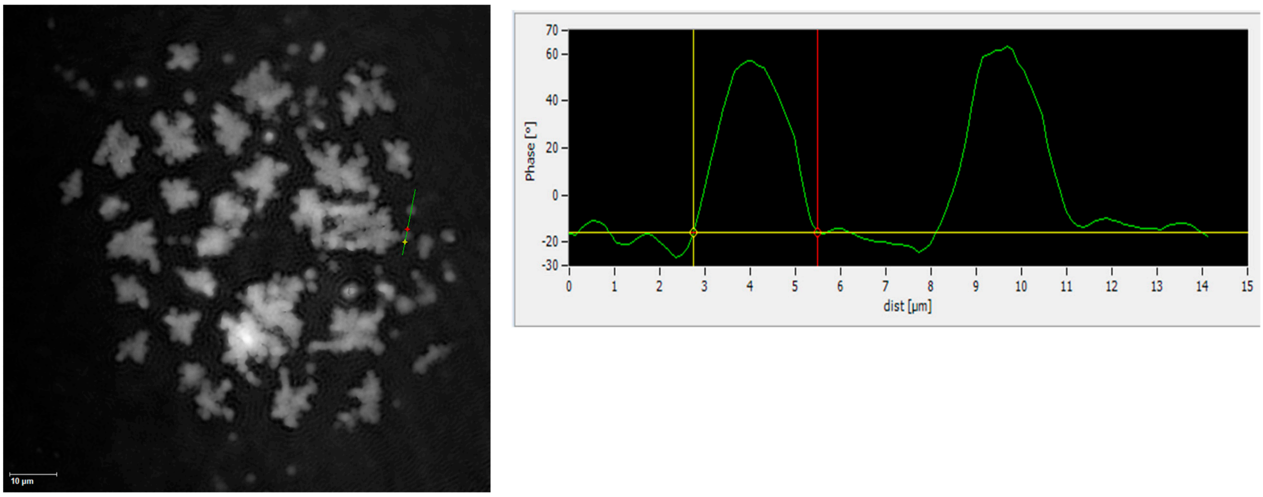

C)

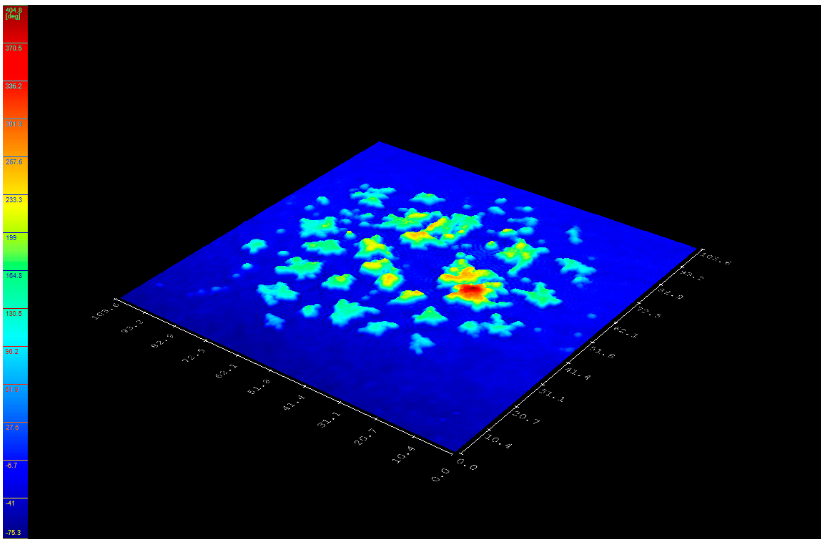

Figure S2 Holographic unwrapped phase image of HAP-particles strewn on a microscope slide -A; the light phase change profile measured along the green line marked in the phase image -B; the computer 3D reconstruction of the holographic image (magnification 50x). The values in degrees on the Y axis correspond to the phase change of the laser beam after passing through the sample -C.
